# The Comorbidities, Radiographic Findings, Age, and Lymphopenia (CORAL) Tool: A Diagnostic Ally for Emergency Physicians Created for the COVID-19 Crisis and Beyond

**DOI:** 10.7759/cureus.41036

**Published:** 2023-06-27

**Authors:** Carlos Castro-Vásquez, Michelle Bass, Gustavo Díaz, Manuel Camargo, Julian Cubillos, Sebastian Alvarez, Luis Garcia-Rairan, Nicolas Sandoval, Adrian Sandoval, Andres M Patiño, Michelle D Lall

**Affiliations:** 1 Department of Emergency Medicine, Trinity Health Livonia Hospital, Michigan State University College of Osteopathic Medicine, Livonia, USA; 2 Department of Medicine, El Bosque University, Bogotá, COL; 3 Institute of Research in Nutrition, Genetics, and Metabolism, El Bosque University, Bogotá, COL; 4 Department of Emergency Medicine, Pontificia Universidad Javeriana, Bogotá, COL; 5 Department of Chemistry, Faculty of Science, National University of Colombia, Bogotá, COL; 6 Department of Emergency Medicine, Emory University School of Medicine, Atlanta, USA

**Keywords:** emergency medicine, intensive care unit, monitoring, screening assessment, clinical decision tool, covid-19

## Abstract

Background: This study aimed to develop a novel clinical approach to predict intensive care unit (ICU) admission and mortality among coronavirus disease 2019 (COVID-19) patients in the emergency department (ED).

Methods: A retrospective cohort study was conducted including adults ≥ 18 years diagnosed with COVID-19 in the emergency department and admitted to the ICU between March and July 2020 in an academic hospital. The outcome variables were mortality and ICU admission. Additional variables that were collected included sex, age, comorbidities, symptom phenotype, and laboratory (lymphopenia) and imaging findings. A logistic regression model was used to construct and validate the risk models.

Results: A total of 808 patients were included in the study; 61.9% were men. The mean age was 57.8 ± 15.9 years, and high blood pressure (HBP) was the most prevalent comorbidity (31.8%). Seventy-six (9.4%) patients were admitted to the ICU. Age ≥ 60 years, chronic obstructive pulmonary disease (COPD), lymphopenia, and imaging findings correlated with mortality. Age ≥ 60 years, lymphopenia (<1,000 cells per microliter), and hypothyroidism correlated with ICU admission. These variables were incorporated into a scoring system (Comorbidities, Radiographic findings, Age, and Lymphopenia (CORAL) tool) to predict mortality and ICU admission.

Conclusions: Our Comorbidities, Radiographic findings, Age, and Lymphopenia (CORAL) tool is a practical tool for different clinical settings independent of access to advanced medical resources or technologies. CORAL is suitable for emergency physicians in low- and middle-income countries.

## Introduction

In December 2019, coronavirus disease 2019 (COVID-19) emerged as a new entity, derived from a previously known virus that caused severe acute respiratory syndrome (SARS virus). COVID-19 was reported as pneumonia of unknown origin on December 31, 2019, that caused a significant number of intensive care unit (ICU) admissions and mortality. By January 7, 2020, this new organism became known as the novel coronavirus (SARS-CoV-2) [1].

Emergency providers (EPs), who acted as frontline personnel since the beginning of the COVID-19 pandemic, are the ones who continuously face challenges and are the main responsible actors in clinical decision-making as to whether patients should be admitted or discharged, even as we enter a post-COVID-19 era. EPs are always aiming at building and improving healthcare for the population in need and are committed to high-quality patient care, teaching, leadership, research, and innovation [2].

The primary organ affected by SARS-CoV-2 is the lung. Patient self-reported symptoms are of great importance in clinical scenarios. Symptoms such as shortness of breath and cough are often related to inflammation, increased risk of respiratory failure, cardiopulmonary arrest, and death [3]. Despite intense and widespread research on prevention measures (e.g., vaccination) as well as treatment of COVID-19-related complications, this disease continues to be an important cause of death with an estimated global excess mortality of 14.91 million based on the 24 months between January 1, 2020, and December 31, 2021 [4].

Among the 50 countries that had the highest incidence of COVID-19, 33 were low- to middle-income countries (LMICs). Most LMICs relied on other countries for vaccine supply due to the lack of resources for developing, testing, and manufacturing vaccines [5]. Colombia is categorized as LMIC and was at a disadvantage in terms of medical resources during the COVID-19 pandemic compared to higher-income countries (HICs).

During the pandemic and the transition to an endemic phase of the disease, a significant number of patients diagnosed with COVID-19 required admission to intensive care units (ICUs). In LMICs, the capacity for critical illness care was limited to approximately 0.5-2.5 beds per 100,000 people. In HICs, bed availability in the ICU was 5-30 per 100,000 people. This big difference revealed that LMICs were unprepared to handle a crisis of overwhelming magnitude such as that of the SARS-CoV-2 pandemic [6]. The combination of insufficient vaccine numbers, scarcity of hospitals and ICU resources, poverty, and social distress became the perfect recipe for chaos, which further debilitated the healthcare infrastructure of LMICs [7].

Based on the latter, there are continuous efforts for the early identification of patients at an increased risk of COVID-19-related complications that may require ICU admission and be at risk of mortality, more so for LMICs. In the present paper, a standardized, integrative, and innovative approach for risk stratification and patient disposition, named Comorbidities, Radiographic findings, Age, and Lymphopenia (CORAL) was developed and applied at the emergency department (ED) of San Rafael Hospital in Bogota, Colombia. CORAL may also assist in identifying patients who can be safely discharged.

## Materials and methods

Design and setting

This is a retrospective cohort study of adults ≥ 18 years old with International Classification of Diseases, 10th Revision, Clinical Modification (ICD-10-CM) diagnosis for COVID-19 in the emergency department and admitted to the ICU from March 1, 2020, to July 31, 2020. The study was conducted at the San Rafael Hospital in Bogota, Colombia, which is a national referral center with an estimated capacity of 150 beds in the emergency department and approximately 40 adult ICU beds. Before doing the data collection, we expressed no conflicts of interest and submitted the clinical research project to the hospital ethics board review. We obtained ethics approval prior to participant recruitment.

Selection of participants

A total sample of 808 patients was selected for the study and included in this analysis. Exposure in our study is SARS-CoV-2-diagnosed patients, which ultimately is associated with ICU admission and death. The sampling method was non-probabilistic. The following inclusion criteria were established: (1) patients must have a laboratory-confirmed SARS-CoV-2 infection, (2) patients must have been ≥18 years old and admitted to the ICU, (3) complete medical records were required, and (4) there should be at least one complete blood count and computed tomography chest imaging. Among the exclusion criteria, the ethical considerations (being a minor) and practical considerations (sudden death or pneumonia of unknown cause) were considered not eligible to be included in the analysis.

Data collection and measurements

To avoid subconscious bias, the collector was blinded to the objectives and hypothesis of the present study. Variables were coded and determined in a coded guide for abstractors. Missing and conflicting data were addressed in cases where there was insufficient or nonhomogeneous information throughout all the clinical records; for this reason, these types of variables were not included in the analysis.

Data collection for this study included all information from electronic clinical records; sex, age, comorbidities, symptom phenotype, imaging findings, past medical history, self-reported symptoms, laboratory values on admission, and chest computed tomography imaging were included for this analysis. Radiographic findings were extracted word by word from the attending radiologist’s report at San Rafael Hospital. The included comorbidities for evaluation based on the frequency of the study sample were high blood pressure (HBP), diabetes mellitus (DM), chronic obstructive pulmonary disease (COPD), hypothyroidism, and obesity.

Statistical analysis, sample size, and power

An estimation for sample size calculation in a cohort study was performed with a β of 80%, α of 5%, a non-exposed percentage of 10%, an odds ratio (OR) of 2, and 30% of missing data. The total sample size was 808 participants. For developing a multivariable logistic prediction model, the sample size was determined with an OR of 2, ∝ of 0.05, power of 0.95, R2 of 0.6, and 20% of loss data. For instance, the database was randomly split into a training set (70%, n = 565) and a testing set (30%, n = 243).

Categorical variables were expressed as frequency or percentages and then compared using χ2 or Fisher’s exact tests. Continuous variables were presented as mean ± standard deviation and then compared using the Mann-Whitney U test or Student’s t-tests. Kolmogorov-Smirnov tests were used for the evaluation of normal distributions.

Logistic regression models were adjusted using mortality and ICU admission as dependent variables. Demographic and clinical variables were included as independent variables. Clinical variables were converted into dichotomous variables based on optimal cutoff values. Independent variables were checked for collinearity and independence. Significant predictors (p < 0.05) were preserved in the final models after a backward selection was performed. Following the principles stated in the TRIPOD checklist, a risk score was developed by assigning each significant variable remaining in the final model. We also established a weighted risk score model based on odds ratios and a second risk score model by adding one point by each variable in the model. The area under the curve (AUC) of the receiver operating characteristic curve, sensitivity, specificity, and Cohen’s kappa were used to evaluate the prediction performance of the model. Confidence intervals at 95% (95% CI) were also reported. Descriptive analysis was performed using Statistical Package for the Social Sciences (SPSS) version 20 (IBM SPSS Statistics, Armonk, NY, USA).

## Results

Patients

A total of 808 patients composed the cohort; 61.9% were men with a mean age of 57.8 ± 15.9 years. Hypertension was the most prevalent comorbidity at 31.9% (n = 258). ICU admission occurred in 9.4% (n = 76) of patients, and 9.2% (n = 74) had a fatal outcome. “Symptom phenotype” (defined as a specific group of symptoms referred by patients and classified into two types: type 1 (respiratory symptoms) and type 2 (patients with myalgia, dyspnea, and diarrhea)) was the only different variable between the training set and the testing set (p < 0.05). Dyspnea is not necessarily due to lung dysfunction. It may arise from multiple causes aside from lung pathology, such as heart failure, anemia, and neurological and psychological causes, to name a few of them. Based on the prior statement, dyspnea is not included in phenotype 1 due to its multicausal nature; it is not solely respiratory in nature.

ICU admission

Seventy-six (9.4%) patients were admitted to the ICU. The main admission criteria were the requirement for airway protection and low oxygen saturation (89.5%). The mean ICU length of stay was 10.5 ± 6.7 days, and the mortality rate of those admitted to the ICU was 59%.

Mortality predictors and ICU admission

A logistic regression model was developed using the training dataset (n = 565). Variables for mortality prediction (n = 4) and ICU admission (n = 3) were identified (Tables 1-3).

**Table 1:** Factors associated with mortality (a) Values ​​presented as frequencies and percentages (%). (b) Values ​​presented as means and standard deviations (±). Hosmer-Lemeshow test: 0.230 HBP: high blood pressure, DM: diabetes mellitus, COPD: chronic obstructive pulmonary disease, URS: upper respiratory symptoms, COVID-19: coronavirus disease 2019, CI: confidence interval, OR: odds ratio

	Vital state		p-value	Logistic regression model	95% CI
	Alive (n = 515)	Dead (n = 47)		OR	
Sex, men (a)	310 (60.2)	32 (68.1)	0.289	3.4	1.5-7.5
Age (b)	56.2 ± 15.2	69 ± 13.7	0.001		
≥60 years (a)	220 (42.7)	37 (78.7)	0.001		
Comorbidities (a)					
HBP	155 (30.1)	18 (38.3)	0.244	3.7	1.5-9.2
DM	94 (18.3)	12 (25.5)	0.222		
COPD	29 (5.6)	10 (21.3)	0.001		
Hypothyroidism	50 (9.7)	10 (21.3)	0.014		
Overweight/obesity	58 (11.3)	5 (10.6)	0.897		
Phenotype (a)					
URS	254 (49.3)	6 (12.8)	0.001		
Myalgia, diarrhea, fever, fatigue	260 (50.5)	40 (85.1)	0.001		
COVID-19 suggestive findings	461 (89.5)	42 (89.4)	0.999		
Image findings (a)					
Patchy bilateral consolidation	33 (7.1)	3 (7.1)	0.999	2.5	1.1-6.0
Linear opacities	41 (8.9)	9 (21.4)	0.026		
Ground-glass opacities	388 (84)	30 (71.4)	0.038		
Lymphocyte count (b)	1096 ± 598	878 ± 597	0.003		
Lymphopenia (a)	266 (51.9)	35 (74.5)	0.003	2.3	1.1-5.0

**Table 2:** Factors associated with ICU admissions (part 1) (a) Values presented as frequencies and percentages (%). (b) Values presented as average and standard deviations (±). Hosmer-Lemeshow test: 0.230 HBP: high blood pressure, DM: diabetes mellitus, COPD: chronic obstructive pulmonary disease, URS: upper respiratory symptoms, ICU: intensive care unit, CI: confidence interval, OR: odds ratio

	ICU admission		p-value	Logistic regression model	
	No	Yes		OR	95% CI
	n = 513	n = 52			
Sex, men (a)	309 (60.2)	35 (67.3)	0.319	2	1.1-3.8
Age (b)	56.7 ± 15.5	62.7 ± 14.2	0.004		
≥60 years (a)	222 (43.3)	35 (67.3)	0.001		
Comorbidities (a)					
HBP	157 (30.6)	16 (30.8)	0.981	2	0.95-4.4
DM	97 (18.9)	9 (17.3)	0.778		
COPD	33 (6.4)	6 (11.4)	0.157		
Hypothyroidism	49 (9.6)	11 (21.2)	0.01		
Overweight/obesity	56 (10.9)	7 (13.5)	0.578		
Phenotype (a)					
URS	245 (47.8)	17 (32.7)	0.038		
Myalgia, diarrhea, fever, fatigue	267 (52)	34 (65.4)	0.066		

**Table 3:** Factors associated with ICU admissions (part 2) (a) Values presented as frequencies and percentages (%). (b) Values presented as averages and standard deviations (±). Hosmer-Lemeshow test: 0.230 HBP: high blood pressure, DM: diabetes mellitus, COPD: chronic obstructive pulmonary disease, URS: upper respiratory symptoms, ICU: intensive care unit, COVID-19: coronavirus disease 2019, CI: confidence interval, OR: odds ratio

	ICU admission		p-value	Logistic regression model	
	No	Yes		OR	95% CI
	n = 513	n = 52			
COVID-19 suggestive findings	458 (89.3)	46 (88.5)	0.856		
Imaging findings (a)					
Bilateral consolidation	33 (7.2)	3 (6.5)	0.999	2.5	1.3-4.8
Linear opacities	42 (9.2)	8 (17.4)	0.113		
Ground-glass opacities	384 (83.7)	35 (76.1)	0.193		
Lymphocyte count (b)	1099 ± 596	861 ± 603	0.001		
Lymphopenia (a)	263 (51.6)	39 (75)	0.001		

Some predictors were common to both outcomes (mortality and ICU admission); however, only mortality contained COPD among its predictors. The goodness of fit was adequate for each model (i.e., mortality or ICU admission).

Risk scores for mortality and ICU admission

After identifying the predictors for ICU admission and mortality, a risk score was developed. The risk scores for predicting mortality ranged from 0 to 10 and from 0 to 4 (lowest to highest risk). The first score was estimated by assigning points according to the odds ratio (OR) of each predictor of mortality; the second score was calculated by assigning 1 point for each of the four predictors of mortality. The risk scores for predicting ICU admission ranged from 0 to 7 and from 0 to 3. ICU admission scores were estimated by assigning points according to the odds ratio of each predictor of ICU admission; the second score was calculated by assigning 1 point for each of the three predictors of ICU admission.

Prediction performance for mortality in the training set yielded an AUC of 0.747 (95% CI: 0.67-0.83, p < 0.001) and 0.67 (95% CI: 0.60-0.75, p < 0.001). In terms of ICU admission, the training dataset of AUC was 0.68 (95% CI: 0.56-0.79, p = 0.001) for the prediction of mortality and 0.60 (95% CI: 0.48-0.72, p < 0.001) for the prediction of ICU admission (Table 4).

**Table 4:** Mortality and ICU admission prediction models Model 1: Point assignment according to OR values (0-10). Model 2: Assignment of 1 point per each variable (0-4). Model A: Point assignment according to OR values (0-7). Model B: Assignment of 1 point per each variable (0-3). SE: standard error, CI: confidence interval, AUC: area under the curve, ICU: intensive care unit

			Cut point	AUC	SE	95% CI
Mortality	Preparation	Model 1	4 or more	0.756	0.037	0.683-0.830
		Model 2	2 or more	0.747	0.039	0.670-0.825
	Validation	Model 1	4 or more	0.699	0.057	0.587-0.811
		Model 2	2 or more	0.676	0.057	0.564-0.789
ICU admission	Preparation	Model A	3 or more	0.679	0.039	0.602-0.756
		Model B	1 or more	0.673	0.04	0.596-0.751
	Validation	Model A	3 or more	0.592	0.063	0.468-0.716
		Model B	1 or more	0.602	0.061	0.482-0.722

The sensitivity and specificity details of the risk scores are shown in Table 5.

**Table 5:** Comparison between prediction models Sen: sensitivity (value and 95% CI), Spe: specificity (value and 95% CI), PPV: positive predictive value, NPV: negative predictive value, ICU: intensive care unit

			Dead	Alive	Agreement	Sen	Spe	PPV	NPV
Model 1	Preparation	Dead	32	155	0.165	79.20%	66.30%	17.10%	96.80%
		Alive	10	305	0.106-0.225	61.5-86.5	61.9-70.5		
	Validation	Dead	16	73	15.4	72.70%	63.50%	18%	95.50%
		Alive	6	127	0.06-0.25	51.9-86.9	56.6-69.9		
Model 2	Preparation	Dead	32	155	0.165	79.20%	66.30%	17.10%	96.80%
		Alive	10	305	0.106-0.225	61.5-86.5	61.9-70.5		
	Validation	Dead	16	73	15.4	72.70%	63.50%	18%	95.50%
		Alive	6	127	0.06-0.25	51.9-86.9	56.6-69.9		
			ICU admission	No ICU admission	Agreement	Sen	Spe	PPV	NPV
Model A	Preparation	ICU admission	41	278	0.07	78.90%	45.50%	12.90%	95.50%
		No ICU admission	11	232	0.03-0.12	66-87.8	41.2-49.8		
	Validation	ICU admission	16	133	0.02	66.70%	39%	10.70%	91.40%
		No ICU admission	8	85	0-0.08	46.7-82	32.8-45.6		
Model B	Preparation	ICU admission	46	357	0.05	88.50%	30%	11.40%	96.20%
		No ICU admission	6	153	0.01-0.08	77-94.6	26.2-34.1		
	Validation	ICU admission	21	170	0.02	87.50%	22%	11%	94.10%
		No ICU admission	3	48	0-0.07	70-95.7	17-28		

## Discussion

After three years since COVID-19 was declared an international public health problem, over six million deaths have been reported worldwide, and eventually, it transitioned into an endemic cause of disease. Depending on individual cases, SARS-CoV-2 infection has a wide range of clinical presentations ranging from asymptomatic infection to severe pneumonia, often refractory to standard measures, followed by multiorgan system failure and death.

Many clinical prediction tools have been used during the COVID-19 pandemic, including A-DROP, CURB-65 (Confusion, Urea, Respiratory rate, Blood pressure, and age ≥ 65), Pneumonia Severity Index (PSI), SMART-COP (Systolic blood pressure, Multilobar infiltrates, Albumin, Respiratory rate, Tachycardia, Confusion, Oxygen, and pH), and National Early Warning Score 2 (NEWS2). Fan et al. [8] compared seven scores and found that A-DROP had a better performance for predicting hospital mortality. However, most of these scores did not consider the patient’s symptoms, additional comorbidities (COPD and hypothyroidism), or chest imaging studies. In contrast, all these factors were included in our CORAL tool and gained relevance in our population, because behavior, management, and prognosis in SARS-CoV-2 pneumonia must be taken into account due to the high variability of COVID-19, compared to bacterial, fungal, or other types of viral pneumonia [9].

Due to the variability in the clinical presentation of COVID-19 and its severity, a clinical prediction tool should assist the physician in both disposition decisions and outcomes. These factors reflect the need for an individualized approach in emergency medicine that takes into consideration specific patient characteristics for clinical decision-making, which will positively impact patient outcomes [10]. Personalized medicine is considered the future of medical practice and healthcare delivery with treatments directed at the patient’s symptoms and adjusted to the patient’s phenotype [11].

Through analysis of the information gathered in the present study, we observed a group of characteristics that were common to most of the patients admitted to the ICU but died by the end of the study. Those variables were analyzed using a multivariable logistic regression model, considering age (>60 years), lymphopenia, comorbidities (COPD and hypothyroidism), and radiographic findings (presence of linear opacities/ground-glass opacities). This analysis resulted in the construction of the present analysis method named “CORAL,” Comorbidities (COPD and hypothyroidism), Radiographic findings (linear opacities/ground-glass opacities), Age > 60 years, and Lymphopenia (<1,000 cells per microliter). CORAL is a potential tool intended to be easily applicable and may overcome a breach to access advanced medical knowledge or technology. CORAL can also be used in different clinical scenarios, making it suitable for LMICs and HICs. The use of CORAL for individualizing the initial clinical and laboratory scenario of patients at high risk of ICU admission and mortality is an example of personalized medicine.

To this date, CORAL has the potential to be a prediction tool that considers patient comorbidities, chest imaging findings, age, and lymphocyte counts. To our knowledge, this is the first study in which hypothyroidism is associated with ICU admission in the context of COVID-19, which might open debate about the contribution of this condition to the management and prognosis of patients diagnosed with COVID-19 [12].

It has been reported that low levels of T3 are associated with the progression and worsening of patients admitted to the ICU due to the low T3 syndrome. Low T3 syndrome is a common condition in patients with acute and critical conditions that is directly associated with systemic inflammation [13]. T3 has a direct association with systemic inflammation and proved to have an anti-inflammatory effect and has been found at low levels in severe COVID-19 cases. Low T3 syndrome might be aggravated in the case of uncontrolled chronic hypothyroidism [14-16]. In multiple population studies, COPD is linked to an increased risk of hospitalization, ICU admission, and mortality, which is in keeping with our results [17-20].

Imaging findings have also been correlated with COVID-19 severity and could be a tool to identify patients who require hospital admission. However, to date, there is a limited number of clinical prediction rules that include chest imaging as one of its components. This study considers imaging findings in conjunction with other individual characteristics of patients (clinical features and laboratory results) to predict the severity of COVID-19. Some imaging-based scores, such as the CT severity score, have been described, but their complexity requires a fully trained and experienced radiologist to interpret the images and calculate the score; these requirements may hinder the universal application of these scores [21,22]. In keeping with most of the published data to date [8,9], in our study, age ≥ 60 years was correlated to increased mortality and ICU admission.

In our results, lymphopenia was a common finding in patients admitted to the ICU and those with a fatal outcome. Other studies have described patients with severe disease and progression who have low lymphocyte counts, especially low CD4+ and CD8+ T cells, with an inverse correlation with SARS-CoV-2 viral load. As demonstrated in our study and previous work [23], in a clinical scenario, in conjunction with the study findings, low lymphocyte counts must be considered a marker of severe disease and increased risk of mortality [24-26].

In a bivariate analysis, self-reported symptoms (most commonly dyspnea, fatigue, myalgia, and diarrhea), defined here as “symptom phenotype,” had a significant correlation with mortality. However, in a multivariate analysis, “symptom phenotype” was not significant for the prediction model. We suggest adding this variable to the score in future studies with a bigger sample size.

Before starting data collection, the project was presented to the ethics board review of San Rafael Hospital. To present a project for a clinical research study, we had to perform an extensive review of the literature, where we found that some symptoms predicted or were associated with disease severity, alongside chest radiography changes. One of the items initially collected in the database was symptoms referred by patients, which were isolated symptom by symptom in the initial analysis. After those initial results, these symptoms (the most common) were grouped into two main groups (phenotypes) as mentioned before. The latter is a novel approach that we based on literature review and study findings, which is believed to be practical and of clinical significance [27-33].

An additional proposed use of the CORAL tool can be considered depending on the patient’s social status and health conditions, and based on their clinical criteria, the EPs may consider admitting or discharging a patient who seems stable but has a high-risk score according to the CORAL tool. In this scenario, patients could be suitable for remote patient monitoring (RPM), aiming for proper management if they show alarming symptoms. Timely RPM may, in turn, optimize and improve the clinical outcomes of patients. It is well known that RPM was used before the COVID-19 pandemic for managing chronic conditions (e.g., diabetes and hypertension) [34]. A cross-sectional study from an RPM clinical program by a New York health system (involving 112 patients with confirmed or suspected COVID-19 diagnoses) showed that RPM is an optimal telemedicine standard to care for COVID-19 patients [35].

COVID-19 is still an important cause of morbidity and mortality worldwide. There is still a need to recognize, evaluate, and monitor patients who require hospitalization and ICU admission early. We argue that the CORAL tool can have a significant impact on decision-making, helping clinicians decide whether a patient should be admitted to the hospital or discharged.

CORAL tool clinical vignette

**Figure 1:**
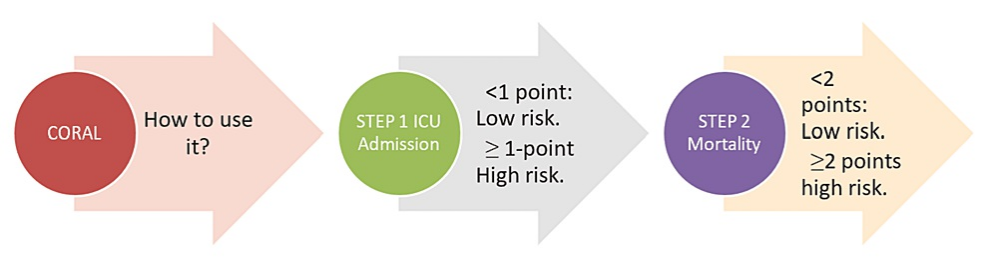
Illustration of CORAL tool application CORAL: Comorbidities, Radiographic findings, Age, and Lymphopenia, ICU: intensive care unit

A brief presentation of a hypothetical clinical scenario where the CORAL tool can be applied is shown in Figure 1.

Mr. X, a 56-year-old male living in a small rural area, presented to the emergency department with a three-day history of dry cough associated with intermittent fevers, sore throat, and anosmia. He has a past medical condition of high blood pressure and gout that are well-controlled. Vital signs show low-grade fever, otherwise within normal limits, and his physical examination is unremarkable. Chest X-ray shows no infiltrates or changes in the lung parenchyma, and blood work on the metabolic panel shows elevated blood cell count with no changes in absolute lymphocyte count and no electrolyte abnormality. Mr. X, who turns out to be SARS-CoV-2-positive by a nasal swab, comes for an emergency consultation alongside his wife and older son. After a thorough assessment, Dr. B finds him to have a low risk for mortality and eventually discharges Mr. X based on clinical judgment and using the CORAL tool.

Limitations

This is a retrospective study, and as such, it has potential information bias. We propose a consecutive study for external validation, as specificity improves upon repetition. Symptom phenotype should be further studied and validated in larger population samples. Additional studies would also likely improve the specificity of the CORAL tool. It is important to mention that the CORAL tool should be ideally used early in the course of the disease, detecting patients at high risk of complications; however, sensitivity is higher when the disease is more severe and evident and may not be applicable in those patients with delayed presentation.

Future perspectives

As we enter a post-COVID-19 pandemic era to an endemic phase, it is important to keep in mind that outbreaks will not become a thing of the past, and as new SARS-CoV-2 variants continue to emerge and circulate, we must learn from the COVID-19 pandemic to be better prepared and ready to deploy massive, coordinated responses in the future. It has been reported that LMICs and HICs were shown to have better outcomes when confronting outbreaks and epidemics by learning and building upon previous experiences to respond to newly emerging infectious diseases [36]. For this reason, we highlight the importance of implementing tools that can be applied, such as the CORAL tool, for future reference.

## Conclusions

CORAL is a potential aid that could easily be applied for different clinical scenarios independent of access to advanced medical resources or technologies. CORAL application in emergency medicine may promote individualized medicine and connection with resources outside of the hospital, including RPM. We showed for the first time that low T3 levels correlated with poor outcomes for COVID-19; thus, we recommend thyroid studies in the initial evaluation of COVID-19 patients. In addition, early intervention and appropriate disposition in patients with COVID-19 presenting to the emergency department are critical to ensure proper resource utilization and ultimately individualized and improved patient care.
